# Supplementary material for: Clinical and Genetic Features of Korean Patients with Achromatopsia
Source: Genes (Basel). 2023 Feb 18;14(2):519. doi: 10.3390/genes14020519 (PMC9957537; doi:10.3390/genes14020519)
Supplement: Supplementary file 1 [file genes-14-00519-s001.zip › genes-2166553-supplementary.pdf]

Table S1. Number of patients in CNGA3 and PDE6C achromatopsia, grouped by structural changes obtained from OCT.

Features		OCT findings			
		**ISe change (−)**	**ISe change (+)**	***p*-value**	**Alternative hypothesis**
Genes with variants, n	*CNGA3*	5	3	0.023 *[,a]	*CNGA3*-ACHM group has higher proportion of normal ISe
	Others	2	10		
	PDE6C	0	8	0.003 *[,b]	*PDE6C*-ACHM group has lower proportion of normal ISe
	Others	7	5		

[a] Boschloo's one-tailed exact test comparing the proportion of patients with normal ISe. The alternative hypothesis of each test is described in the right-most column. OCT findings were derived from the date of the most recent OCT images. Abbreviations: OCT, optical coherence tomography; ISe, inner segment of ellipsoid zone.

Table S2. Results of pairwise comparison of one-sided, exact tests comparing features associated with structural changes.

**Alternative Hypothesis** The group of patients with the causative gene in the row has a higher proportion of normal ISe on the OCT than the groups of patients with the gene in the column.			
	CNGB3	*PDE6C*	*GNAT2*
CNGA3	0.094	0.005 *	1.0
CNGB3		N/A	1.0
PDE6C			1.0

[a] Boschloo's one-tailed exact test comparing the proportion of patients with normal ISe. OCT findings were derived from the date of the most recent OCT images. Abbreviations: OCT, optical coherence tomography; ISe, inner segment of ellipsoid zone.

Table S3. Number of patients with structural changes obtained from OCT.

	**OCT Findings**		*p*-value
Genes with variants	ISe change (−)	ISe change (+)	
CNGA3	5	3	0.047 *[,a]
Others	2	10	
CNGB3	0	2	0.557 [a]
Others	7	11	
PDE6C	0	8	0.007 *[,a]
Others	7	5	
GNAT2	2	0	0.121 [a]
Others	5	13	

[a] Boschloo's two-sided exact test, OCT findings, and age were derived from the date of the most recent OCT images. Abbreviations: OCT, optical coherence tomography; ISe, inner segment of ellipsoid zone.
